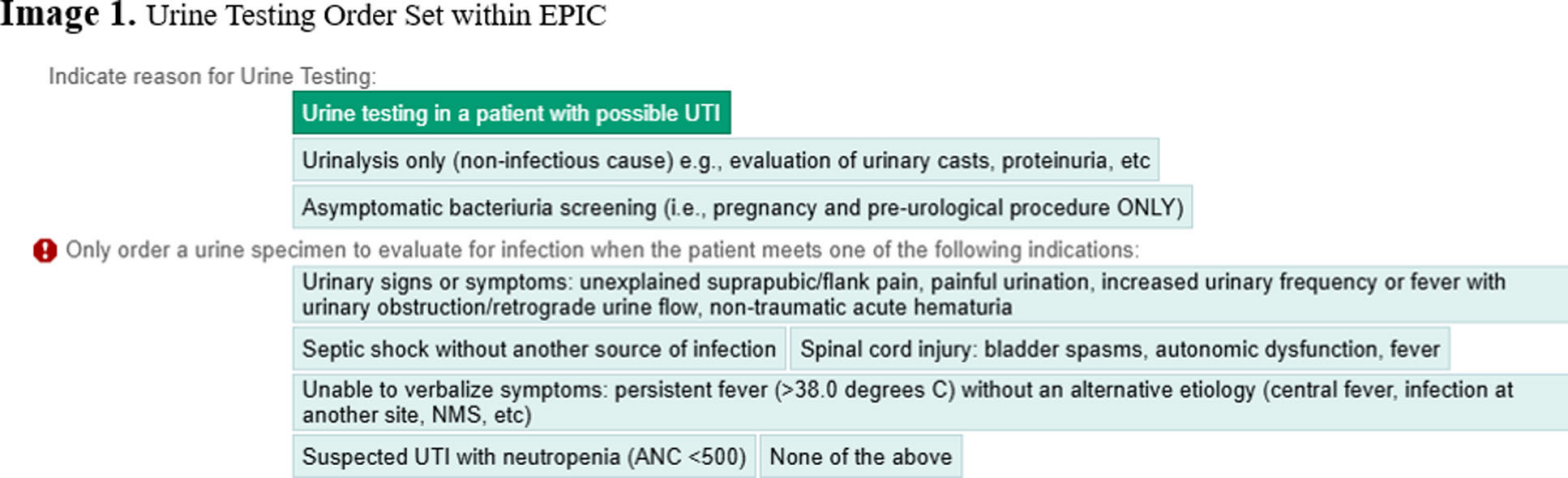# 195 Quantitative Parvovirus B19 PCR and Clinical Correlates in Children: Implications for Infection Prevention

**DOI:** 10.1017/ash.2026.10584

**Published:** 2026-06-23

**Authors:** Mario Luis Valdez Imbert, Allyson Larcena Tipgos, Majd Alsoubani, Maureen Campion, Rachel Erdil, Shira Doron, Gabriela Andujar-Vazquez, Kap Sum Foong

**Affiliations:** 1 Tufts Medical Center; 2 Tufts Medicine; 3 Dartmouth Hitchcock Medical Center; 4 Tufts Medical Center, Tufts University School of Medicine

## Abstract

**Background** Inappropriate urine culturing is a driver of unnecessary antibiotic use and treatment of asymptomatic bacteriuria. Diagnostic stewardship interventions, including clinical decision support(CDS), can reduce urine culture utilization; however, their impact on downstream testing patterns and antibiotic prescribing remains limited. In August 2024, Tufts Medical Center implemented an indication-based CDS requiring providers to select a guideline-concordant indication when ordering inpatient urinalysis(UA), UA with reflex to culture(UARC), or urine culture alone(UCx) We evaluated the impact of this intervention on urine testing utilization, ordering appropriateness, and associated antibiotic prescribing. Methods We conducted an interrupted time series analysis of monthly urine testing rates from August 2023 through July 2025, with CDS implementation in August 2024. The primary outcome was utilization of urine testing, measured as rates of UA, UARC, and UCx per 1000 patient days. Segmented regression models with a normal distribution and identity link were used to estimate baseline trends, immediate level changes at implementation, and post-intervention trend differences(ITS). As a secondary outcome, we retrospectively reviewed a random sample of 153 adult patients post-implementation UARC orders to assess urine testing appropriateness and antibiotic prescribing based on clinical documentation and predefined institutional criteria(Image 1). Results During the study, 124,762 urine tests were ordered across 225,614 census days. Prior to CDS implementation, UA, UARC, and UCx rates showed no significant secular trends(Figure 1-3). Following implementation, UARC and UCx rates showed immediate and significant level decrease. UARC utilization increased post-implementation but remained below pre-intervention levels, while UCx rates showed a sustained reduction without significant post-intervention trend change. In contrast, UA rates increased immediately after implementation, followed by a non-significant decline. Table 1 summarizes effect estimates. Of 153 audited UARC orders, 46.4% originated from the emergency department (ED), 41.2% from intensive care unit and 12.4% from inpatient floor units. Urinary signs or symptoms were the most frequently selected indication for UARC, particularly in the ED(95.8%). Documentation supported appropriate UARC testing in only 9.9% of ED orders, 19.2% of floor orders, and 27.8% of ICU orders. Antibiotics were initiated for presumed urinary tract infection(UTI) in 29 patients(19%), of whom 9(5.9%) had documented UTI. Conclusion Indication-based urine testing CDS intervention produced immediate and intended changes in testing utilization (decreased UARC and Ucx, and increased UA), followed by partial attenuation of the UARC effect over time. Post-implementation chart audit demonstrated persistent gaps in documentation-supported appropriateness and antibiotic initiation, underscoring the need for additional targeted stewardship interventions beyond CDS alone.

**Figure:**
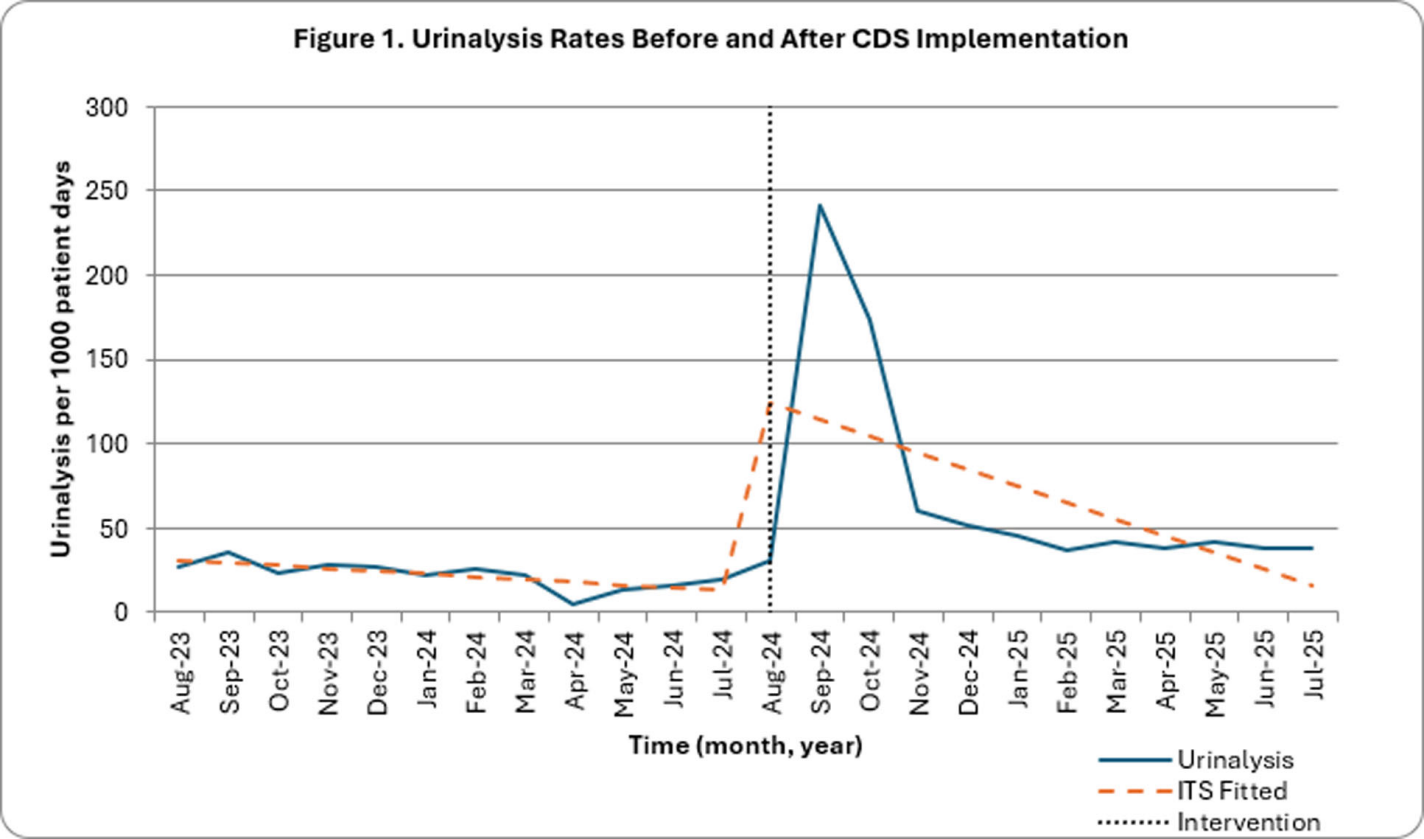

**Figure:**
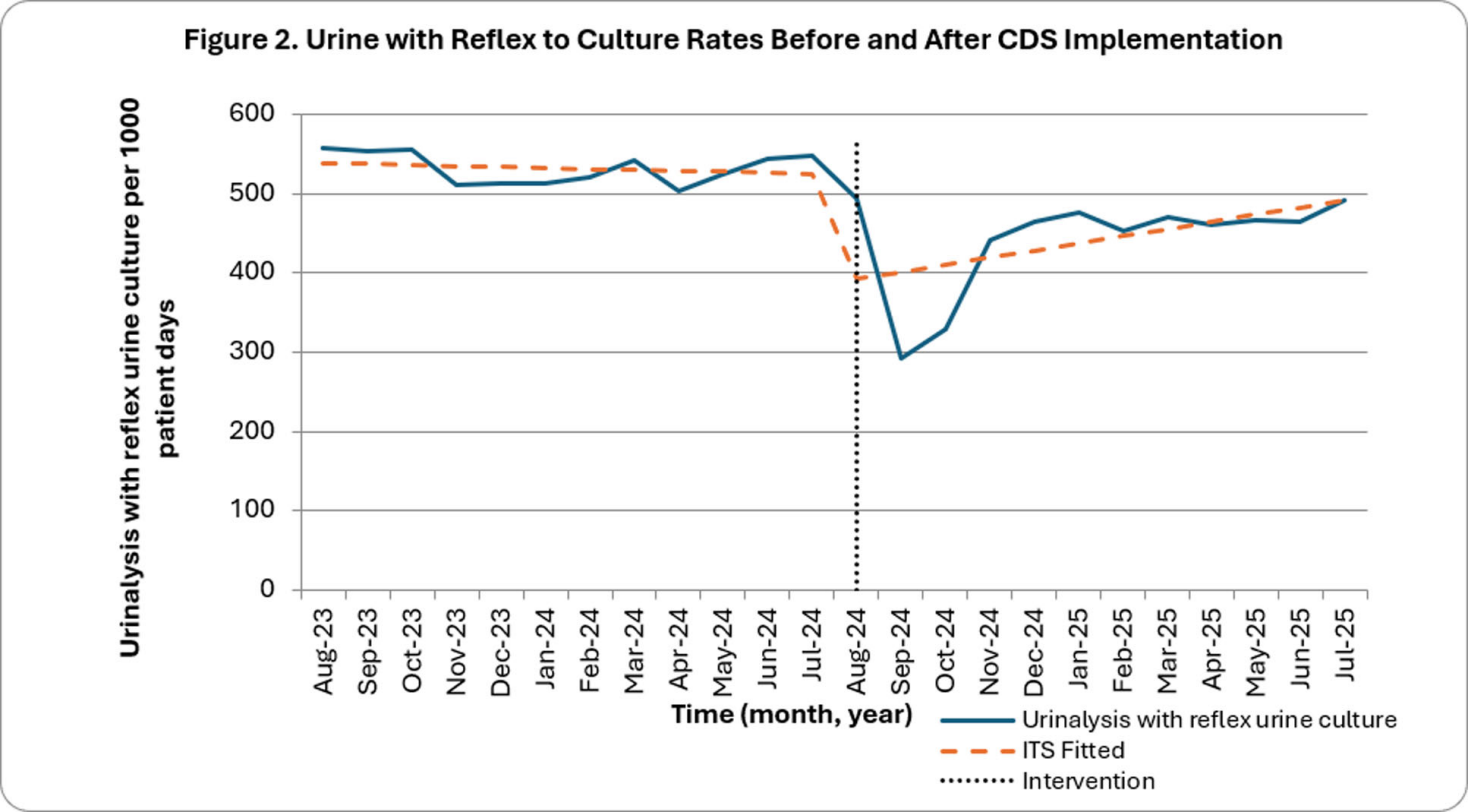

**Figure:**
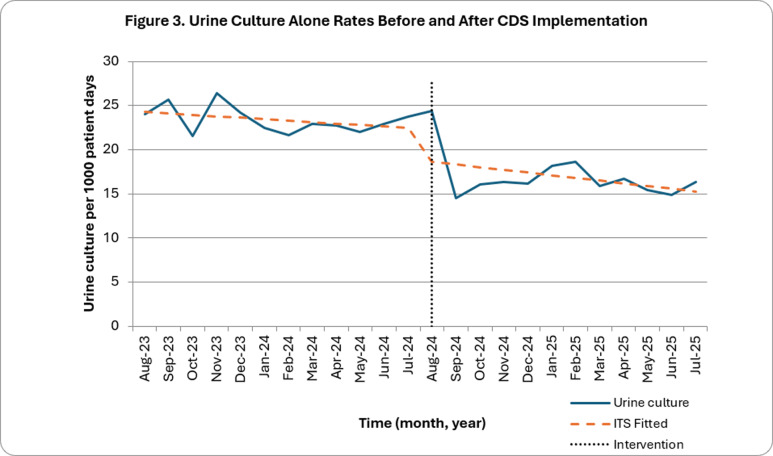

**Figure:**
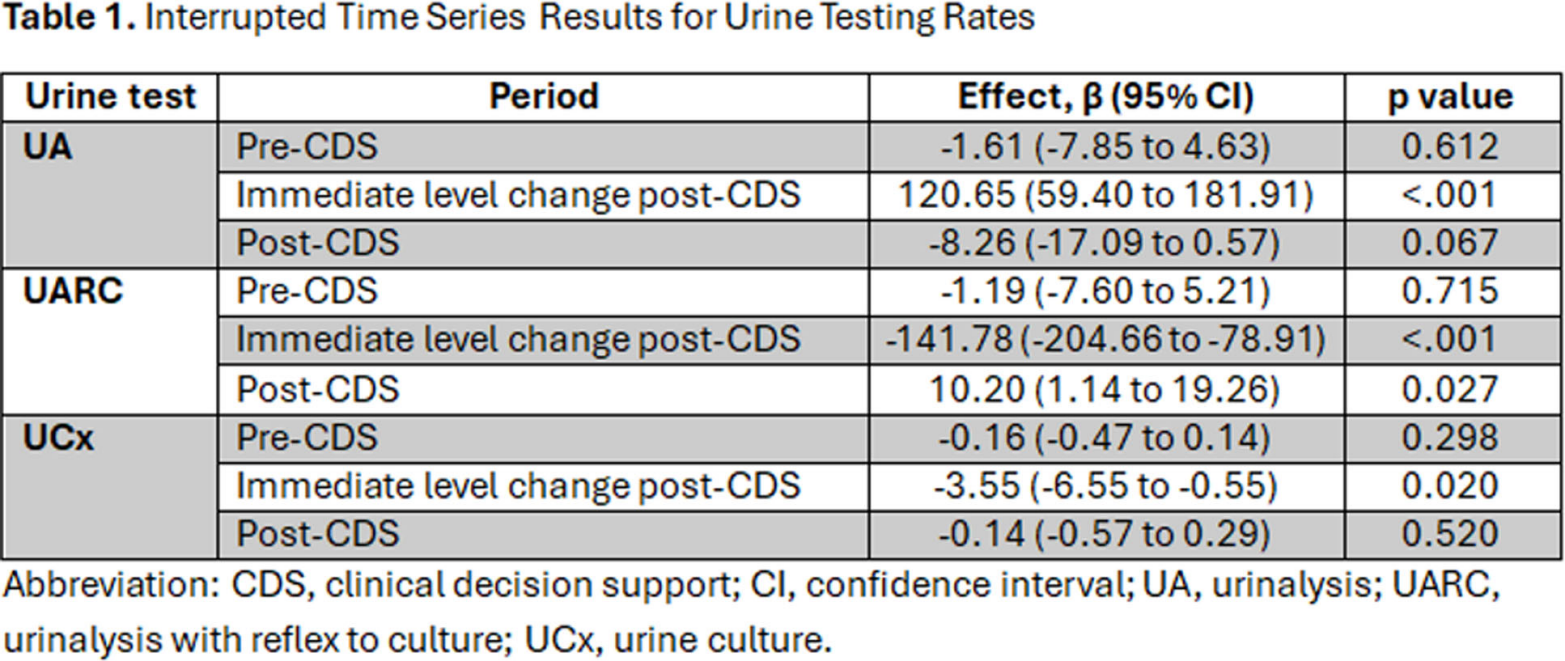

**Figure:**